# Characterization of the m^6^A regulators’ landscape highlights the clinical significance of acute myocardial infarction

**DOI:** 10.3389/fimmu.2024.1308978

**Published:** 2024-03-20

**Authors:** Peng Chao, Xueqin Zhang, Lei Zhang, Yong Wang, Miriban Wusiman, Gulizere Aimaijiang, Xiaoyang Chen, Yining Yang

**Affiliations:** ^1^ Department of Cardiology, People’s Hospital of Xinjiang Uygur Autonomous Region, Urumqi, China; ^2^ Xinjiang Key Laboratory of Cardiovascular Homeostasis and Regeneration Research, Urumqi, Xinjiang Uygur Autonomous Region, China; ^3^ Department of Nephrology, People’s Hospital of Xinjiang Uygur Autonomous Region, Urumqi, China; ^4^ Department of Endocrinology, People’s Hospital of Xinjiang Uygur Autonomous Region, Urumqi, China

**Keywords:** acute myocardial infarction, N^6^-methyladenosine, immune cell infiltration, ferroptosis, oxidative stress, ALKBH5

## Abstract

**Objective:**

Acute myocardial infarction (AMI) is a severe cardiovascular disease that threatens human life and health globally. N^6^-methyladenosine (m^6^A) governs the fate of RNAs via m^6^A regulators. Nevertheless, how m^6^A regulators affect AMI remains to be deciphered. To solve this issue, an integrative analysis of m^6^A regulators in AMI was conducted.

**Methods:**

We acquired transcriptome profiles (GSE59867, GSE48060) of peripheral blood samples from AMI patients and healthy controls. Key m^6^A regulators were used for LASSO, and consensus clustering was conducted. Next, the m^6^A score was also computed. Immune cell infiltration, ferroptosis, and oxidative stress were evaluated. *In-vitro* and *in-vivo* experiments were conducted to verify the role of the m^6^A regulator ALKBH5 in AMI.

**Results:**

Most m^6^A regulators presented notable expression alterations in circulating cells of AMI patients versus those of controls. Based on key m^6^A regulators, we established a gene signature and a nomogram for AMI diagnosis and risk prediction. AMI patients were classified into three m^6^A clusters or gene clusters, respectively, and each cluster possessed the unique properties of m^6^A modification, immune cell infiltration, ferroptosis, and oxidative stress. Finally, the m^6^A score was utilized to quantify m^6^A modification patterns. Therapeutic targeting of ALKBH5 greatly alleviated apoptosis and intracellular ROS in H/R-induced H9C2 cells and NRCMs.

**Conclusion:**

Altogether, our findings highlight the clinical significance of m^6^A regulators in the diagnosis and risk prediction of AMI and indicate the critical roles of m^6^A modification in the regulation of immune cell infiltration, ferroptosis, and oxidative stress.

## Introduction

Acute myocardial infarction (AMI) remains a severe cardiovascular disease that threatens human life and health across the globe, which is caused by the rupture or erosion of vulnerable atherosclerotic plaques with superimposed thrombosis, leading to coronary occlusion and progressive cell death in areas of low perfusion (1). The proportion of young adults among AMI patients has increased in recent years (2). The widespread adoption of reperfusion treatment and improved antithrombotic strategy have remarkably improved AMI patients’ prognosis (3). Nonetheless, prompt revascularization may result in reperfusion damage, thus lowering the clinical benefit of revascularization (4). There are currently no effective therapeutic strategies for myocardial ischemia/reperfusion damage. In addition, AMI is still the leading cause of chronic heart failure (5). Thus, it is of significance to discover novel biomarkers with strong sensitivity and specificity for diagnosing AMI (6).

N^6^-methyladenosine (m^6^A) represents the most studied RNA epi-transcriptomic modification, which affects the diverse mRNA metabolism traits and the biogenesis of non-coding RNAs. The m^6^A modification process is governed by three types of m^6^A regulators: methyltransferases, m^6^A-binding proteins, and demethylases (7). Recent investigations demonstrate the significance of m^6^A modification in AMI. For instance, alterations in m^6^A modification result in heart failure progression through modulation of translation (8). Deletion of m^6^A methyltransferase METTL3 triggers heart regeneration and repair following myocardial damage (9). m^6^A demethylase ALKBH5 deficiency leads to post-ischemic angiogenesis through post-transcriptionally stabilizing WNT5A (10). Ferroptosis is a form of programmed cell death triggered by iron-dependent accumulation of lipid hydroperoxide (11). Experimental evidence proves that mitigation of ferroptosis alleviates AMI in murine models (12). m^6^A methyltransferase METTL14 motivates doxorubicin-induced ferroptosis of cardiomyocytes (13). AMI is usually correlated to increased oxidative stress and inflammation, and both result in disease progression and are modulated by m^6^A modification (14). Thus, the current research implemented an integrative analysis of m^6^A regulators in blood samples of AMI patients. We built an m^6^A regulator gene model for diagnosing AMI and predicting disease risk, proposed a novel m^6^A-based classification of AMI, and unveiled the relationships of m^6^A modification with immune cell infiltration, ferroptosis, and oxidative stress, thus potentially improving the clinical management of AMI.

## Materials and methods

### Acquisition of AMI datasets

AMI datasets were queried from the Gene Expression Omnibus (GEO), which is a public functional genomics data repository (https://www.ncbi.nlm.nih.gov/geo/). After screening, the GSE59867 (15) and GSE48060 (16) datasets were included in the current study. The GSE59867 dataset comprised gene expression profiles of peripheral blood samples from 111 AMI patients and 46 healthy controls. The GSE48060 dataset covered transcriptome data of blood samples from 31 AMI patients and 21 controls. Both datasets were based on the Affymetrix platform. The Affymetrix data were preprocessed using the affy package (17). The GSE59867 dataset acted as the training set, while the GSE48060 dataset acted as the testing set.

### Collection of m^6^A regulators

We gathered the list of 25 m^6^A regulators (**Supplementary Table 1**). The chromosomal location of m^6^A regulators was visualized with the RCircos package (18). The transcript levels of m^6^A regulators were compared in AMI relative to healthy controls in the GSE59867 dataset. Relationships between regulators were inferred utilizing Pearson’s correlation analysis.

### Generation of an m^6^A-based gene signature for AMI diagnosis

Through univariate and multivariate logistic regression analyses, m^6^A regulators with *p <*0.05 were screened out. To avoid overfitting of the data, we further selected the least absolute shrinkage and selection operator (LASSO) to reduce dimensionality. The LASSO coefficient of each gene was calculated through ten-fold cross-validation, and the key m^6^A regulators with non-zero LASSO coefficients were selected to develop an m^6^A gene regulator model utilizing the glmnet package (19). The risk score was computed by linearly combining the key m^6^A regulators weighted by the corresponding coefficients. Receiver operator characteristic curves (ROCs) were plotted to investigate the diagnostic efficacy of the risk score and each key m^6^A regulator.

### Nomogram establishment

To predict the risk of AMI, we incorporated the expression level of the 10 key m^6^A gene regulators selected by LASSO analysis to construct a nomogram through the rms package. Then, the consistency between the actual observation and predicted probability was assessed through calibration curves (by a bootstrap method with 1,000 resamples). In addition, the clinical utility of the nomogram was investigated by using decision curve analysis together with clinical impact curves.

### Consensus clustering analysis

Consensus clustering was implemented for building an m^6^A regulator-based classification using the “ConsensusClusterPlus” package, with Euclidean distance and the K-means clustering approach (20). A total of 1,000 bootstraps were performed using the “K-means” algorithm with “Euclidean” distance. Each bootstrap consisted of 80% of the AMI samples. The range of cluster numbers, *k*, was set from two to nine, and the optimal value for *k* was determined based on the cumulative distribution function (CDF) curve and the CDF delta area curve. To visually represent the cluster membership, a consensus matrix heatmap was generated. Moreover, the difference in transcriptome profiling was visualized via the principal component analysis (PCA).

### Immune cell infiltration

Utilizing the GSVA package with default parameters (21), the single-sample gene set enrichment analysis (ssGSEA) algorithm was executed to estimate the abundance of each immune cell type in each sample based on the transcriptome data and specific immune cell markers (22).

### Selection of m^6^A cluster-relevant genes

Genes with differential expression between m^6^A clusters (m^6^A cluster A vs. m^6^A cluster B, m^6^A cluster A vs. m^6^A cluster C, m^6^A cluster B vs. m^6^A cluster C) were screened using the limma package (23). The intersecting genes with adjusted *p <*0.05 were selected as m^6^A cluster-relevant genes, as previously described (24).

### Functional enrichment analysis

Enrichment analysis of Gene Ontology (GO) terms together with the Kyoto Encyclopedia of Genes and Genomes (KEGG) pathways of m^6^A cluster-relevant genes was executed utilizing the clusterProfiler package (25).

Gene set enrichment analysis (GSEA) was utilized to select significant functional gene sets with the fgsea package (26), with the “h.all.v7.0.symbols” of the molecular signatures database used as the predefined hallmark gene set (27). Briefly, all the expressed genes were ranked according to the stat values from the differential expression analysis. Next, all ensemble IDs of the differentially expressed gene list were mapped to the “org.Hs.eg.db” annotation file to generate a map table. The enrichment score (ES) was computed through the permutation test for each gene set. Adjusted *p <*0.05 was utilized as the cutoff for interpreting the results.

### Definition of the m^6^A score

We extracted m^6^A cluster-relevant genes to define the m^6^A score for PCA. The formula for computing the m^6^A score is as follows: m^6^A score = ∑ (PC1i + PC2i), where PC1i and PC2i denote the transcript levels of m^6^A cluster-relevant genes in principal components 1 and 2. The Sankey diagram was drawn to illustrate the relationships between m^6^A clusters, gene clusters, and m^6^A scores using the ggalluvial package.

### Cell culture

H9C2 cells were cultivated in high-glucose Dulbecco’s modified Eagle’s medium (SEVEN, Beijing, China) plus 10% fetal bovine serum (HyClone, UT, USA), 100 U/ml of penicillin, and 100 µg/ml of streptomycin in a humidified atmosphere with 5% CO_2_ at 37°C. To build a hypoxia/reoxygenation (H/R) cell model, when the confluence reached 80%~90%, H9C2 cells were exposed to hypoxia (oxygen deprivation, 0.5%) for 6 h and were subsequently cultured in the normal oxygen condition for 12 h (28, 29). To inhibit ALKBH5 expression, H9C2 cells were pretreated with 50 μM of the ALKBH5 inhibitor IOX1 (Selleck, Shanghai, China) for 2 h (4) or transfected with siRNAs against ALKBH5 (si-ALKBH5) (GenePharma, Shanghai, China) for 24 h via Lipofectamine RNAiMAX (Invitrogen, CA, USA).

Neonatal rat cardiomyocytes (NRCMs) were procured from the cardiac tissues of Sprague–Dawley rats aged between 1 and 3 days, sourced from Beijing HFK Bioscience Co., Ltd., China. In brief, ventricular tissues were sectioned and subjected to enzymatic digestion using 0.08% trypsin, repeated 10–12 times, followed by cell collection via centrifugation at 60*g* for 8 min. The harvested cells were then suspended in a suitable plating medium and incubated for 1 h to facilitate the removal of non-cardiomyocytes. Subsequently, the isolated cardiomyocytes were transferred into Dulbecco’s modified Eagle’s medium (DMEM) enriched with 10% newborn calf serum (NBCS) and 0.1 mM of 5-bromodeoxyuridine (Brdu). These cells were then plated at a concentration of 1 × 10^6^ cells per dish in 35 mm dishes coated with gelatin. After a 24-h incubation period in fresh culture medium, the cells were prepared for further experimental analysis.

### Animal experiment

The experimental protocols involving animals in this study received approval from the Experimental Animal Ethics Committee at the People’s Hospital of Xinjiang Uygur Autonomous Region, under the approval number SYDW2023110315. Male C57BL/6J mice, aged 7 weeks, were acquired from Beijing HFK Bioscience Co., Ltd., Beijing, China. All procedures related to animal surgery and care adhered to the NIH’s Guidelines for the Care and Use of Laboratory Animals.

Following a week of acclimatization, the mice, each weighing around 25 g, were divided into four groups at random: control, sham, acute myocardial infarction (AMI), and AMI+rAAV-ALKBH5. To establish the AMI model, we performed ligation of the left anterior descending (LAD) coronary artery. The process involved anesthetizing the mice with pentobarbital sodium (60 mg/kg, intraperitoneally), intubation, and mechanical ventilation, followed by the permanent occlusion of the LAD. Indicators of successful AMI model creation included pallor in the myocardium near the ligation site and ST-segment elevation on the electrocardiogram. The sham group underwent identical surgical procedures, excluding the ligation of the LAD.

For the AMI+rAAV-ALKBH5 group, we utilized an adeno-associated virus serotype 9 (AAV9) vector, equipped with a cytomegalovirus (CMV) promoter, which carried the ALKBH5 gene sequence (termed AAV-ALKBH5). We administered these vectors to C57BL/6 mice through tail vein injections. Each mouse received a dose of 2.5 × 10^11^ vector genomes (vg).

### IHC staining

For immunohistochemical (IHC) analysis, cardiac tissue sections underwent xylene-based deparaffinization and sequential ethanol rehydration. Antigen unmasking followed the protocols specified in the Antibody Manual. To inhibit endogenous peroxidase, sections were treated with 3% hydrogen peroxide for 15 min and then blocked using 5% goat serum for half an hour. Primary antibody (1:300; Beyotime, Wuhan, China) incubation was carried out at 4°C overnight within a moisture-controlled environment, succeeded by an hour-long exposure to HRP-linked secondary antibodies at ambient temperature. Signal development was achieved using a DAB Kit (ZSGB-Bio, Beijing, China), adhering to the provided guidelines. The process concluded with hematoxylin counterstaining of the sections.

### Immunofluorescence

Cells were seeded on a 24-well plate and cultivated until the confluence reached 70%~80%. Cells were washed with PBS three times, fixed with 4% paraformaldehyde (Sangon, Shanghai, China) for 15 min at room temperature, with subsequent permeation with 0.5% Triton® X-100 (Sigma, Shanghai, China) for 20 min. Normal goat serum (Beyotime, Shanghai, China) was added to the cells and closed at room temperature for an hour. The cells were incubated with primary antibody against ALKBH5 (Proteintech, Wuhan, China) and cTNT (Proteintech, Wuhan, China) overnight for 4°C and Alexa Fluor 488-conjugated AffiniPure goat anti-rabbit IgG (Proteintech) for 1 h at 37°C. The cell nucleus was stained with DAPI (Sigma) away from the light for 5 min. Photographs were acquired utilizing a fluorescence microscope (Zeiss, Baden-Wurtberg, Germany).

The procedure for immunofluorescence (IF) staining of cardiac tissue sections paralleled that of IHC, with the distinction that after antigen retrieval, sections were blocked using 5% goat serum, followed by overnight incubation with primary antibodies (Proteintech, Wuhan, China). Subsequently, the sections were treated with Alexa Fluor-tagged secondary antibodies for 1 h at ambient temperature and then mounted in a medium infused with DAPI (Sigma).

### TUNEL staining

Cell apoptosis was measured through the TUNEL apoptosis detection kit (AtaGenix, Wuhan, China). H9C2 cells and NRCMs were inoculated on a 24-well plate. Until the confluence was 70%~80%, cells were fixed with 4% paraformaldehyde (Sangon) for 30 min at room temperature, and permeated with 0.1% Triton® X-100 (Sigma) for 5 min. Cells were administrated with 50 µl of the TUNEL mixture in the dark humified chamber for an hour at 37°C. Next, cells were washed with PBS two times and treated with 0.05 μg/μl of DAPI (Sigma) away from the light for 10 min. Images were acquired by a fluorescence microscope (Zeiss).

### Reactive oxygen species detection

Intracellular reactive oxygen species (ROS) were measured by the ROS detection kit (Beyotime) based on the DCFH-DA staining method. H9C2 cells and NRCMs were seeded on a 24-well plate (3 × 10^5^ cells/well) for 24 h. Cells were washed twice with PBS and exposed to 1 ml of DCFH-DA (10 µM) at 37°C for 20 min away from the light. Subsequently, serum-free DMEM was adopted for the removal of excessive DCFH-DA. Photographs were captured under a fluorescence microscope (Zeiss).

### Western blot

Samples comprising both heart tissues and cells were gathered, followed by the preparation of their lysates. Proteins, in consistent amounts ranging from 40 to 60 μg, were separated via SDS/PAGE and subsequently transferred onto PVDF membranes. These membranes were initially blocked using a 5% milk solution in Tris-buffered saline containing Tween 20 at ambient temperature for 2 h. This was followed by an overnight incubation at 4°C with primary antibodies (ALKBH5: 1:1,000, Abcam, Ab195377; Bcl-2: 1:2,000, Abcam, Ab194583; Bax: 1:10,000, Abcam, Ab32503, United Kingdom). Subsequently, the membranes were exposed to secondary antibodies (1:20,000; Proteintech, SA00001-2) linked to horseradish peroxidase for 1 h at room temperature, and detection was carried out using a chemiluminescence detection system (GE, Amersham Imager 680RGB, United States). The grayscale intensity of each blot was quantified using ImageJ software (provided by the National Institutes of Health, Bethesda, MD, USA), with band intensities normalized against the reference protein GAPDH (1:20,000; Proteintech, 10494-1-AP) or the aggregate target protein.

### Masson’s trichrome staining and hematoxylin and eosin staining

Heart tissue samples were processed by fixing in 4% paraformaldehyde for a duration of 48 h, followed by dehydration and embedding in paraffin. Subsequently, they were sectioned into continuous slices with a thickness of 5 μm, suitable for histological analysis. To assess the extent of fibrosis in the left ventricle (LV), Masson’s trichrome staining was employed, as detailed in prior studies. The fibrosis level in the LV was calculated as the total collagen area within the LV divided by the LV’s total tissue area, multiplied by 100%. Additionally, the presence of inflammatory cells was evaluated using hematoxylin and eosin (H&E) staining. This involved staining with hematoxylin for 3 min, followed by a 3-min eosin counterstain. After treatment with xylene and mounting, the pathological alterations in the cardiac sections were examined using an Olympus optical microscope.

### Statistical analysis

For continuous variables, the Student’s *t*-test or the Mann–Whitney *U* test was utilized for inferring the differences between two groups, with one-way analysis of variance or the Kruskal–Wallis test for comparison between multiple groups. Categorical data were expressed as numbers (or percent), with the chi-square test or Fisher’s exact test for comparison. Pearson’s or Spearman’s test was utilized for relationships between parameters. R packages (version 3.6.1) were applied for statistical analysis. Two-sided *p <*0.05 was regarded as statistical significance.

## Results

### Expression alterations of m^6^A regulators in AMI patients’ peripheral blood samples

**Figure 1:**
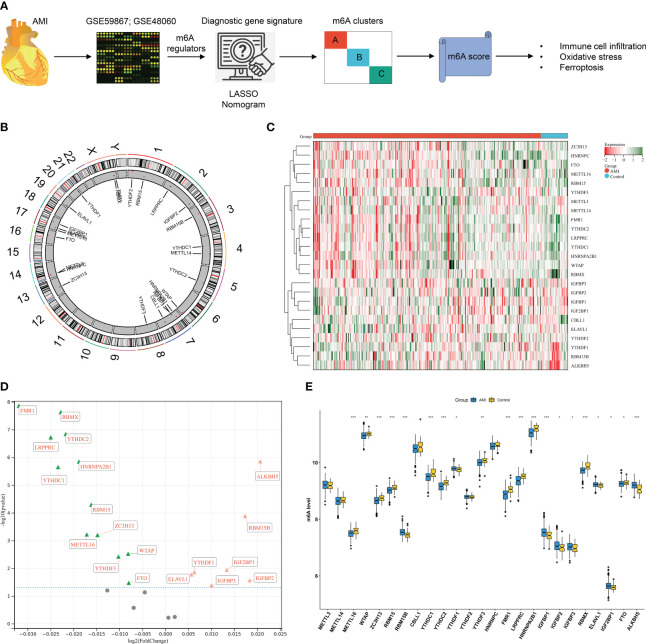
Expression alterations of N^6^-methyladenosine (m^6^A) regulators in acute myocardial infarction (AMI) patients’ peripheral blood samples in the GSE59867 dataset. **(A)** The workflow of the current study. **(B)** Details of the genomic location of m^6^A gene regulators were visualized through a circos plot. **(C)** Heatmap of the transcript expression level of each m^6^A gene regulator in peripheral blood samples from AMI patients and healthy controls. **(D)** Volcano plot of the 25 gene regulator genes: the red triangle represents upregulation in the AMI tissue, the green triangle represents upregulation in the control tissue, and the gray circle denotes no significance between AMI and control tissues. **(E)** Comparison of the expression of m^6^A regulators in AMI patients relative to healthy controls. *p < 0.05; **p < 0.01; ***p < 0.001.

The workflow of the current study is illustrated in **Figure 1A**. We presented an integrative analysis of m^6^A regulators in AMI. The genomic location of each m^6^A regulator is presented in **Figure 1B** and **Supplementary Table 1**. The deregulation of m^6^A regulators was investigated in circulating cells of AMI patients relative to that of healthy controls (**Figures 1C, D**). Specifically, ELAVL1, YTHDF1, IGF2BP1, IGFBP2, RBM15B, IGFBP1, and ALKBH5 displayed significant upregulation in AMI, with downregulation of FMR1, LRPPRC, YTHDC1, RBMX, HNRNPA2B1, YTHDC2, RBM15, CBLL1, METTL16, ZC3H13, YTHDF3, WTAP, and FTO (**Figure 1E**; **Table 1**), indicating that deregulated m^6^A regulators might participate in AMI pathogenesis.

**Table 1:** Deregulation of m^6^A regulators in circulating cells from AMI patients versus controls.

Gene name	Log fold change	Average expression	*t*	*p*	Adjusted *p*	*B*
RBMX	−0.15456	9.73495	−6.72743	5.34E−11	1.34E−09	14.4739
FMR1	−0.19817	8.881158	−6.15456	1.69E−09	2.11E−08	11.12968
ALKBH5	0.131046	9.192855	5.659209	2.73E−08	2.28E−07	8.44076
YTHDC1	−0.1549	9.503309	−5.47348	7.40E−08	4.62E−07	7.482658
YTHDC2	−0.13865	9.167662	−5.16432	3.65E−07	1.83E−06	5.950051
LRPPRC	−0.16321	9.368208	−4.94326	1.09E−06	4.55E−06	4.902572
HNRNPA2B1	−0.14511	11.06645	−4.73791	2.91E−06	1.04E−05	3.966322
RBM15	−0.10141	9.029523	−4.14771	4.03E−05	0.000126	1.477095
METTL16	−0.08946	7.50878	−3.80748	0.00016	0.000396	0.181181
RBM15B	0.090261	7.536696	3.785619	0.000174	0.000396	0.101468
IGFBP1	0.126172	7.528592	3.817964	0.000154	0.000396	0.219585
ZC3H13	−0.08916	8.661571	−3.23333	0.001315	0.002739	−1.76881
YTHDF1	0.042779	9.788286	2.757222	0.00607	0.011673	−3.15521
YTHDF3	−0.07109	10.00372	−2.72084	0.006768	0.012085	−3.25241
CBLL1	−0.09226	10.49531	−2.65774	0.008151	0.013585	−3.41807
WTAP	−0.06175	10.95786	−2.61469	0.009235	0.014407	−3.52892
IGFBP2	0.089491	7.071325	2.594136	0.009797	0.014407	−3.58121
FTO	−0.05129	9.260473	−2.39625	0.016977	0.023579	−4.06425
ELAVL1	0.036409	9.2241	2.260885	0.02425	0.031908	−4.37316
IGF2BP1	0.052229	5.650048	2.078633	0.038225	0.047782	−4.76134
IGFBP3	0.04878	7.023094	1.809108	0.071111	0.084656	−5.2767
METTL14	−0.04139	8.647685	−1.40866	0.159636	0.181405	−5.91212
HNRNPC	−0.03291	10.58945	−1.31483	0.189247	0.205703	−6.03836
METTL3	0.013093	9.207396	0.449258	0.653465	0.680693	−6.79419
YTHDF2	0.004037	8.790888	0.282243	0.777889	0.777889	−6.85478

### Generation of an m^6^A regulator-based gene signature for diagnosing AMI

**Figure 2:**
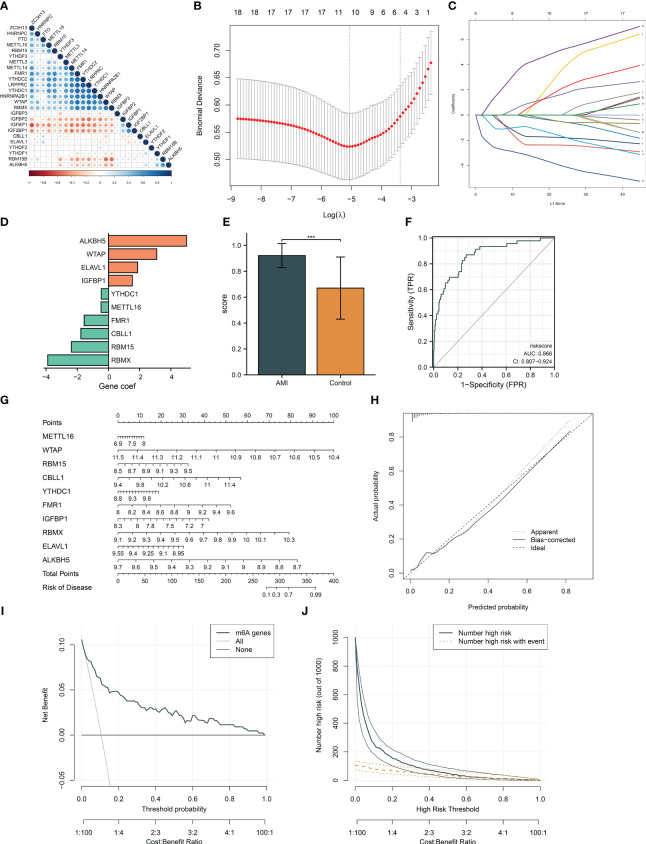
Generation of an m^6^A regulator gene model and a nomogram for diagnosing AMI and risk prediction in the GSE59867 dataset. **(A)** Correlation analysis between m^6^A regulators. The red dot represents a positive association; the blue dot represents a negative association. The bigger the circle, the stronger the association. **(B)** Ten cross-validation analysis for selecting key m^6^A regulators in the least absolute shrinkage and selection operator (LASSO) regression model. **(C)** The coefficient profiling of the LASSO. **(D)** The LASSO coefficient of each key m^6^A gene regulator in the LASSO-derived signature. **(E)** Differences of the m6A risk score between AMI patients and controls. **(F)** ROCs for assessing the diagnostic efficacy of risk score. **(G)** Definition of a nomogram based on key m^6^A gene regulator expression for risk prediction. **(H)** Calibration curve analysis to estimate the relationship of the nomogram predicted with actual probability. **(I)** Decision curve analysis for investigating the clinical net benefit of the nomogram. **(J)** Evaluation of the clinical impact of the nomogram. ***p < 0.001.

At the transcript level, we focused on the notably positive and negative interactions between m^6^A regulators in AMI (**Figure 2A**). Based on univariate and multivariate logistic regression results, WTAP, RBMX, and ALKBH5 were independent factors of AMI risk (**Table 2**). LASSO was executed to select the key m^6^A regulators. Consequently, 10 key m^6^A regulators were determined, comprising METTL16, WTAP, RBM15, CBLL1, YTHDC1, FMR1, IGFBP1, RBMX, ELAVL1, and ALKBH5 (**Figures 2B, C**). The m^6^A regulator-based gene signature was then generated, following the formula: risk score = (−3.891500067) * RBMX transcript level * + (−2.376689271) * RBM15 transcript level + (−1.76826797) * CBLL1 transcript level + (−1.557858912) * FMR1 transcript level + (−0.473279382) * METTL16 transcript level + (−0.461816131) * YTHDC1 transcript level + (1.528727298) * IGFBP1 transcript level + (1.872962542) * ELAVL1 transcript level + (3.096662537) * WTAP transcript level + (5.00949281) * ALKBH5 transcript level (**Figure 2D**). A higher risk score was observed in AMI patients relative to healthy controls (**Figure 2E**). ROCs proved that the m^6^A regulator-based risk score can accurately diagnose AMI (**Figure 2F**).

**Table 2:** Univariate and multivariate logistic regression analyses of m^6^A regulators.

	Univariate analysis		Multivariate analysis	
	OR (95% CI)	*p*	OR (95% CI)	*p*
METTL16	0.016 (0.002–0.142)	2.00e−04	NA	
WTAP	0.066 (0.008–0.517)	0.0097	6.527e+02 (2.993e+00–1.423e+05)	0.018
ZC3H13	0.043 (0.006–0.302)	0.0015	NA	
RBM15	0.011 (0.001–0.099)	<0.0001	NA	
RBM15B	65.168 (7.009–605.964)	2.00E−04	NA	
CBLL1	0.151 (0.037–0.623)	0.0089	NA	
YTHDC1	0.004 (0.000–0.034)	<0.0001	NA	
YTHDC2	0.004 (0.000–0.037)	<0.0001	NA	
YTHDF1	78.913 (3.534–1,761.953)	0.0058	NA	
YTHDF3	0.063 (0.008–0.470)	0.007	NA	
FMR1	0.004 (0.000–0.026)	<0.0001	NA	
LRPPRC	0.014 (0.002–0.086)	<0.0001	NA	
HNRNPA2B1	0.016 (0.003–0.097)	<0.0001	NA	
IGFBP1	18.011 (3.884–83.529)	2.00e−04	NA	
IGFBP2	7.283 (1.578–33.613)	0.0109	NA	
RBMX	0.000 (0.000–0.005)	<0.0001	5.000e−03 (0.000e+00–2.980e−01)	0.011
ELAVL1	33.425 (1.620–689.782)	0.0231	NA	
IGF2BP1	8.323 (1.119–61.903)	0.0385	NA	
FTO	0.068 (0.007–0.624)	0.0174	NA	
ALKBH5	368.645 (39.422–3447.278)	<0.0001	1.256e+03 (2.291e+01–6.886e+04)	0

OR, odds ratio; CI, confidence interval; NA, not available.

### Establishment of an m^6^A regulator-based nomogram for risk prediction

For facilitating clinical application, the current study generated a nomogram comprising key m^6^A regulators for predicting the risk of AMI (**Figure 2G**). The calibration curves demonstrated a high consistency between the nomogram prediction and actual observation (**Figure 2H**). In accordance with the decision curve analysis, the nomogram possessed higher net benefits for identifying AMI (**Figure 2I**). Additionally, clinical impact curves were drawn to assess the clinical utility of the nomogram. The results demonstrated that the nomogram-predicted outcome coincided well with the actual outcome (**Figure 2J**).

### Expression alterations of key m^6^A regulators and their relationships with ferroptosis and oxidative stress and assessment of their diagnostic performance

**Figure 3:**
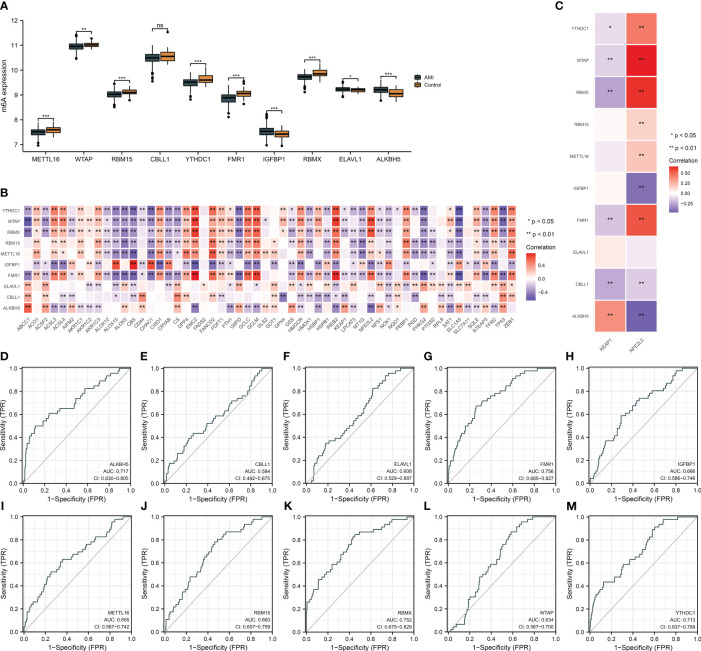
Expression alterations of key m^6^A regulators and their relationships with ferroptosis and oxidative stress and assessment of their diagnostic performance in the GSE59867 dataset. **(A)** Differences in the transcript level of each key m^6^A regulator in AMI and controls. **(B)** Heatmap illustrating the relationship of key m^6^A regulators with ferroptosis genes. Red, positive association; blue, negative association. **(C)** Association between key m^6^A regulators and oxidative stress mediators (NFE2L2 and KEAP1). **(D–M)** ROCs for evaluating the diagnostic ability of each key m^6^A regulator (ALKBH5, CBLL1, ELAVL1, FMR1, IGFBP1, METTL16, RBM15, RBMX, WTAP, and YTHDC1) in AMI. *p < 0.05; **p < 0.01; ***p < 0.001; ns: *p* > 0.05 NS means no significant.

Circulating key m^6^A regulators were deregulated in AMI relative to controls (**Figure 3A**). Evidence proves that m^6^A modification regulates ferroptosis in heart diseases (11, 13). Herein, we noticed that key m^6^A regulators were remarkably associated with most ferroptosis genes in AMI (**Figure 3B**). Previous research unveiled that m^6^A-modified oxidative stress facilitates myocardial ischemia/reperfusion damage (30, 31). NRF2 and its cytoplasmic repressor KEAP1 act as the main regulators of oxidative stress (32). Key m^6^A regulators were observably linked with NFE2L2 and KEAP1 in AMI (**Figure 3C**). ROCs demonstrated that key m^6^A regulators have a conspicuous performance in diagnosing AMI (**Figures 3D-M**). The outstanding diagnostic efficacy of each key m^6^A regulator was also verified in the GSE48060 dataset (**Supplementary Figures 1A–J**).

### Classification of AMI patients into three m^6^A clusters

**Figure 4:**
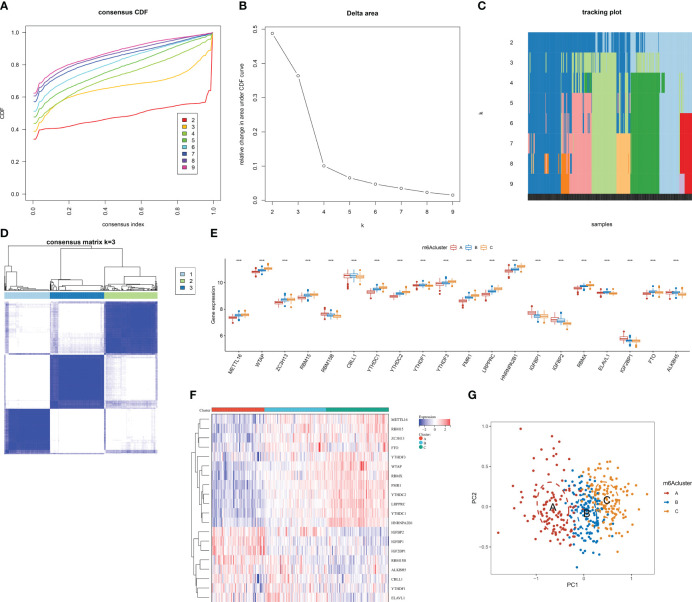
Identification of the three m^6^A clusters associated with AMI in the GSE59867 dataset. **(A)** Consensus clustering cumulative distribution function curve from *k* = 2 to *k* = 9. **(B)** Relative change value in the area under the CDF curve for *k* = 2 to *k* = 9. **(C)** Item tracking plot for *k* = 2 to *k* = 9. **(D)** Consensus matrix at *k* = 3 based on the transcriptome profiling of m^6^A regulators. **(E)** Differences in the transcript level of each m^6^A regulator between m^6^A clusters. **(F)** Heatmap illustrating the transcriptome profiles of m^6^A regulators in each m^6^A cluster. **(G)** PCA plots visualizing the transcriptome differences among the three m^6^A clusters based on m^6^A regulators. ***p < 0.001.

In accordance with the transcriptome profiles of m^6^A regulators, the present research implemented consensus clustering analysis among AMI patients. When *k* = 3, the CDF curve displayed the smallest descending slope (**Figure 4A**). The area under the CDF curve exhibited the lowest decrease when *k* = 4 (**Figure 4B**). Item and consensus matrix plots revealed that AMI patients were obviously stratified into three m^6^A clusters (**Figures 4C, D**). Based on the above evidence, the optimal *k* value was set as 3. Diverse transcript levels of m^6^A regulators were investigated across m^6^A clusters, reflecting the unique m^6^A modification phenotype of each cluster (**Figures 4E, F**). PCA proved the discrepancy in transcriptome profiling among the three m^6^A clusters (**Figure 4G**).

### Three m^6^A clusters with diverse immune cell infiltration, ferroptosis, and oxidative stress traits in AMI

**Figure 5:**
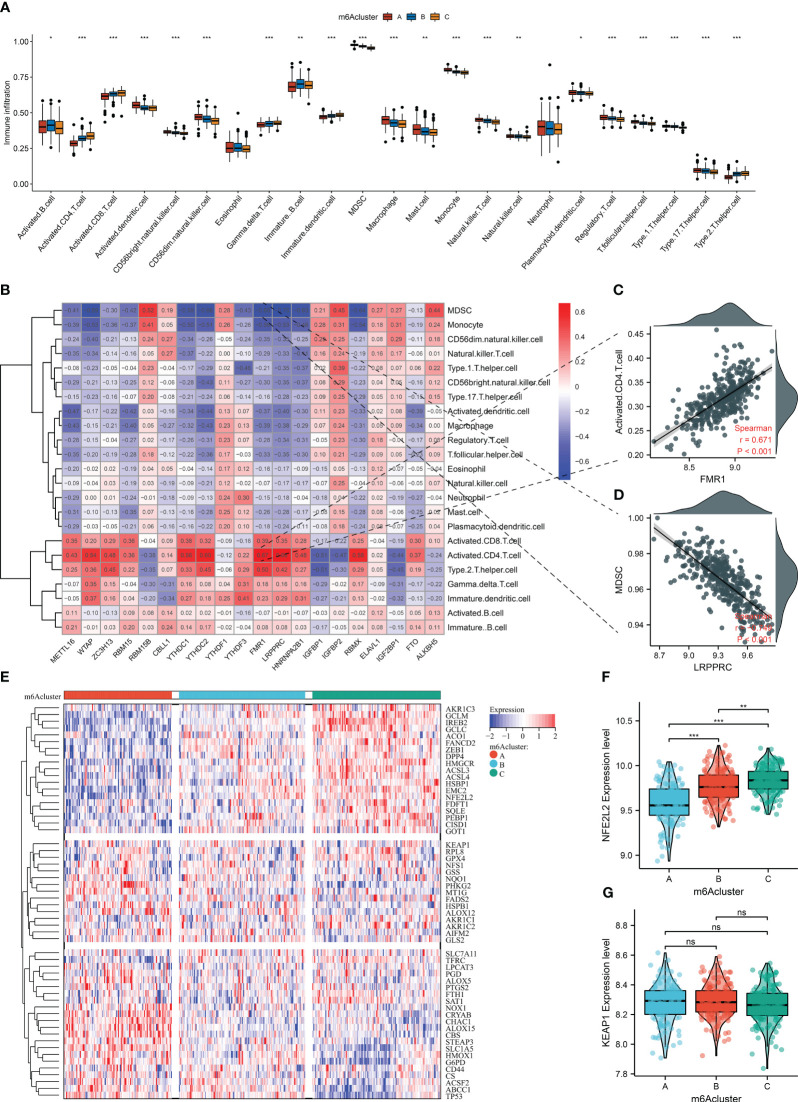
The three m^6^A clusters with diverse immune cell infiltration, ferroptosis, and oxidative stress traits in AMI from the GSE59867 dataset. **(A)** Differences in the infiltration of immune cells between m^6^A clusters. **(B)** Correlation heatmap showed the relationship between m^6^A regulators and the abundance of immune cells. **(C)** Scatter plots of the correlation between FMR1 and activated CD4^+^ T cells. **(D)** Scatter plots illustrating the association of LRPPRC and MDSCs. **(E)** Heatmap of the transcript level of each ferroptosis gene across m^6^A clusters. **(F, G)** Comparison of the transcript levels of oxidative stress mediators (NFE2L2 and KEAP1) between the three m^6^A clusters. *p < 0.05; **p < 0.01; ***p < 0.001; ns: *p* > 0.05 NS means no significant.

Next, the widespread discrepancy in the abundance of most immune cell types was found across the three m^6^A clusters (**Figure 5A**). Each m^6^A regulator was markedly correlated to immune cell infiltration (**Figure 5B**). Notably, FMR1 displayed the strongest positive association with activated CD4^+^ T cells, with the strongest negative relationship between LRPPRC and MDSCs (**Figures 5C, D**). We also focused on the unique ferroptosis features in each m^6^A cluster based on the diverse transcript levels of ferroptosis genes (**Figure 5E**). NFE2L2 presented the highest transcript level in m^6^A cluster C, followed by clusters B and C (**Figure 5F**). No notable discrepancy in KEAP1 level was investigated across m^6^A clusters (**Figure 5G**). Thus, three m^6^A clusters possessed diverse immune cell infiltration, ferroptosis, and oxidative stress traits.

### Selection of m^6^A cluster-relevant genes

**Figure 6:**
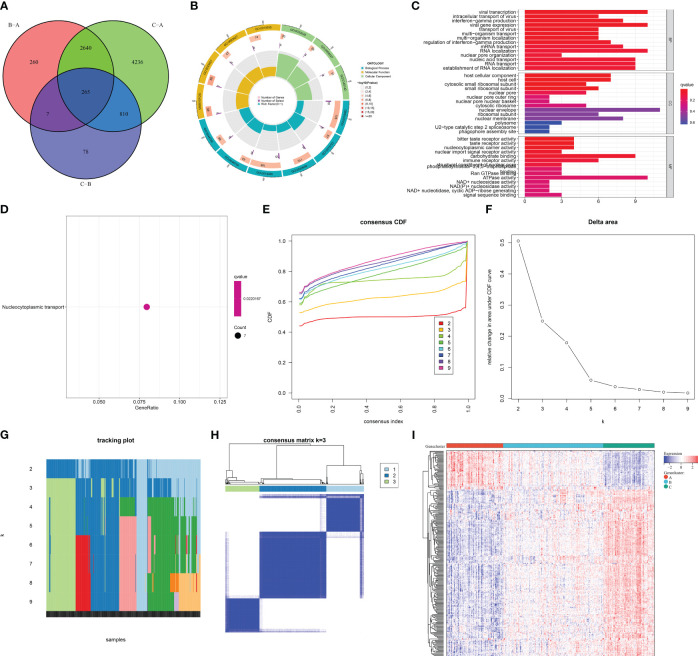
Selection of m^6^A cluster-relevant genes and construction of m^6^A gene clusters in AMI patients from the GSE59867 dataset. **(A)** Venn diagram illustrating the m^6^A cluster-relevant genes through intersecting differentially expressed genes between m^6^A clusters. **(B, C)** The enriched GO terms by m^6^A cluster-relevant genes. **(D)** The enriched one KEGG pathway by m^6^A cluster-relevant genes. **(E)** Consensus clustering cumulative distribution function curve from *k* = 2 to *k* = 9. **(F)** Relative change value in the area under the CDF curve for *k* = 2 to *k* = 9. **(G)** Item tracking plot for *k* = 2 to *k* = 9. **(H)** Consensus matrix at *k* = 3 based on the transcriptome profiling of m6A cluster-relevant genes across distinct m6A gene clusters. **(I)** Heatmap of the transcript levels of m^6^A cluster-relevant genes across distinct m^6^A gene clusters.

To further unveil the m^6^A modification mechanisms, genes with differential expression between m^6^A clusters were selected with adjusted *p <*0.05. After intersecting, 265 m^6^A cluster-relevant genes were eventually acquired (**Figure 6A**; **Supplementary Table 2**). In accordance with the GO annotation results, m^6^A cluster-relevant genes presented notable enrichment of viral infection and mRNA metabolism processes (**Figures 6B, C**). In addition, nucleocytoplasmic transport was prominently linked with m^6^A cluster-relevant genes (**Figure 6D**). These findings proved the functional importance of m^6^A cluster-relevant genes in AMI.

### Construction of the three m^6^A gene clusters across AMI patients

Based on the m^6^A cluster-relevant genes, consensus clustering was executed across AMI patients. The CDF curve presented the smallest descending slope when *k* = 3 (**Figure 6E**). When *k* = 4, the area under the CDF curve had the lowest decrease (**Figure 6F**). Item and consensus matrix plots demonstrated that AMI patients were clearly classified into three m^6^A gene clusters (**Figures 6G, H**). Altogether, the optimal *k* value was 3. In **Figure 6I**, each m^6^A gene cluster presented unique transcript levels of m^6^A cluster-relevant genes.

### The heterogeneity in m^6^A regulators, immune cells, ferroptosis, and oxidative stress among distinct m^6^A gene clusters

**Figure 7:**
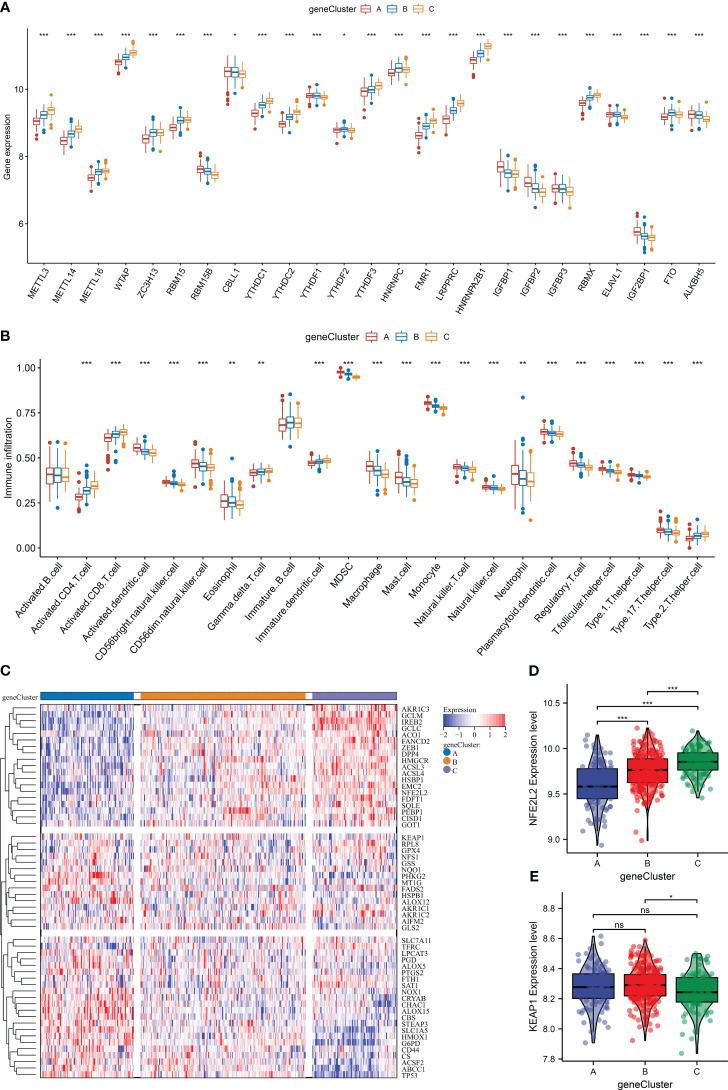
The heterogeneity in m^6^A regulators, immune cells, ferroptosis, and oxidative stress among distinct m^6^A gene clusters in the GSE59867 dataset. **(A)** Differences in the transcript level of each m^6^A regulator across m^6^A gene clusters. **(B)** Comparison of the abundance of immune cells between m^6^A gene clusters. **(C)** Heatmap of the transcript levels of ferroptosis genes in each m^6^A gene cluster. **(D, E)** Differences in the transcript levels of oxidative stress regulators (NFE2L2 and KEAP1) between m^6^A gene clusters. *p < 0.05; **p < 0.01; ***p < 0.001; ns: *p* > 0.05. NS means no significant.

As expected, the three m^6^A gene clusters presented prominent differences in transcript levels of m^6^A regulators, proving the distinct m^6^A modification patterns (**Figure 7A**). In addition, heterogeneity in the infiltration of most immune cells was found across m^6^A regulators (**Figure 7B**). Next, the current study investigated ferroptosis activity among m^6^A gene clusters. As illustrated in **Figure 7C**, ferroptosis genes displayed unique transcript levels in each gene cluster. m^6^A gene cluster A possessed the lowest level of NFE2L2, followed by clusters B and C (**Figure 7D**). Also, lower KEAP1 expression was investigated in m^6^A gene cluster C (**Figure 7E**). The above evidence proved the heterogeneity in m^6^A regulators, immune cells, ferroptosis, and oxidative stress among diverse m^6^A gene clusters.

### Definition of the m^6^A score system for AMI

**Figure 8:**
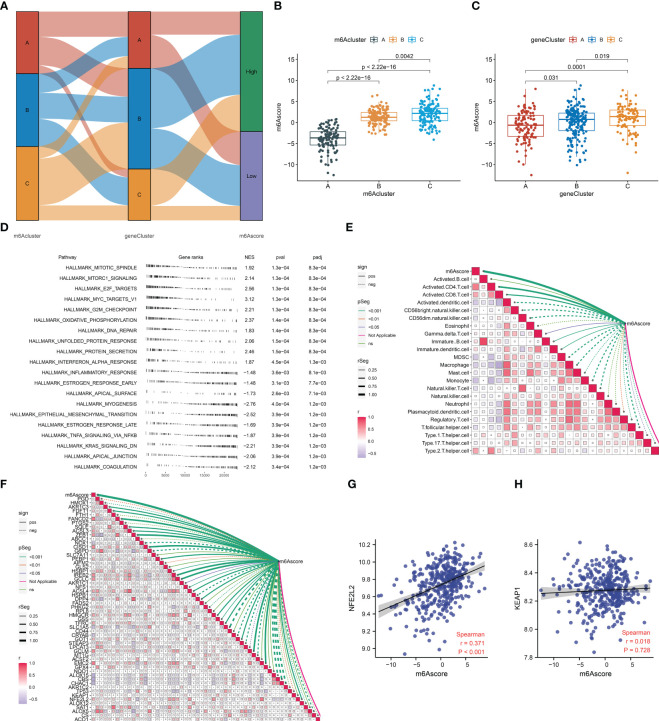
Definition of the m^6^A score and its association with the hallmark gene set, immune cells, ferroptosis, and oxidative stress in AMI from the GSE59867 dataset. **(A)** Sankey diagram depicting the relationship among the m^6^A clusters, m^6^A gene clusters, and m^6^A scores. **(B, C)** Differences in the m^6^A score between m^6^A clusters and m^6^A gene clusters. **(D)** The enriched hallmark gene set based on the m^6^Ascoe. Each black line represents a gene. The *X*-axis denotes gene rank and ranked genes from the differentially expressed gene list based on their expression value at the top or bottom of the predefined gene list. The *Y*-axis denotes the hallmark gene set. The three columns on the right are the normalized enrichment score (NES) and *p*-value (pval) together with the adjusted *p*-value (padj). **(E)** Association between the m^6^A score and the abundance of immune cells. The solid line denotes a positive correlation, while the dashed line indicates a negative correlation. The thicker the outer border of the box, the stronger the correlation. **(F)** Relationship between the m^6^A score and the transcript level of each ferroptosis gene. The solid line denotes a positive correlation, while the dashed line indicates a negative correlation. The thicker the outer border of the box, the stronger the correlation. **(G, H)** Scatter plots illustrating the correlation of the m^6^A score with the transcript level of NFE2L2 and KEAP1.

To quantify the m^6^A modification in AMI, PCA was adopted for the extracted m^6^A cluster-relevant genes (**Figure 8A**). A remarkable difference in the m^6^A score was noticed across the three m^6^A clusters (**Figure 8B**). m^6^A cluster C presented the highest m^6^A score, followed by cluster B, and cluster A had the lowest score. A similar m^6^A score pattern was observed among the three m^6^A gene clusters (**Figure 8C**). Hence, the m^6^A score can reflect the m^6^A modification in AMI.

### Association between the m^6^A score and hallmark gene set, immune cells, ferroptosis, and oxidative stress in AMI

The mechanisms underlying the m^6^A score were further probed during AMI. The significant hallmark gene sets linked with the m^6^A score were selected utilizing GSEA. In **Figure 8D**, proliferation-, inflammatory response-, and fibrosis-relevant gene sets were remarkably associated with the m^6^A score. Notably, the m^6^A score presented positive relationships with activated CD4^+^ and CD8^+^ T cells and immature dendritic cells, with negative associations with MDSCs, macrophages, monocytes, natural killer T cells, and type 1 helper cells (**Figure 8E**). Moreover, prominent relationships between the m^6^A score and most ferroptosis genes were observed (**Figure 8F**). For two key mediators of oxidative stress (NFE2L2 and KEAP1), the m^6^A score exhibited a significantly positive correlation to NFE2L2 (**Figures 8G, H**).

### Therapeutic targeting of ALKBH5 alleviates apoptosis of H/R-induced H9C2 cells and NRCMs

**Figure 9:**
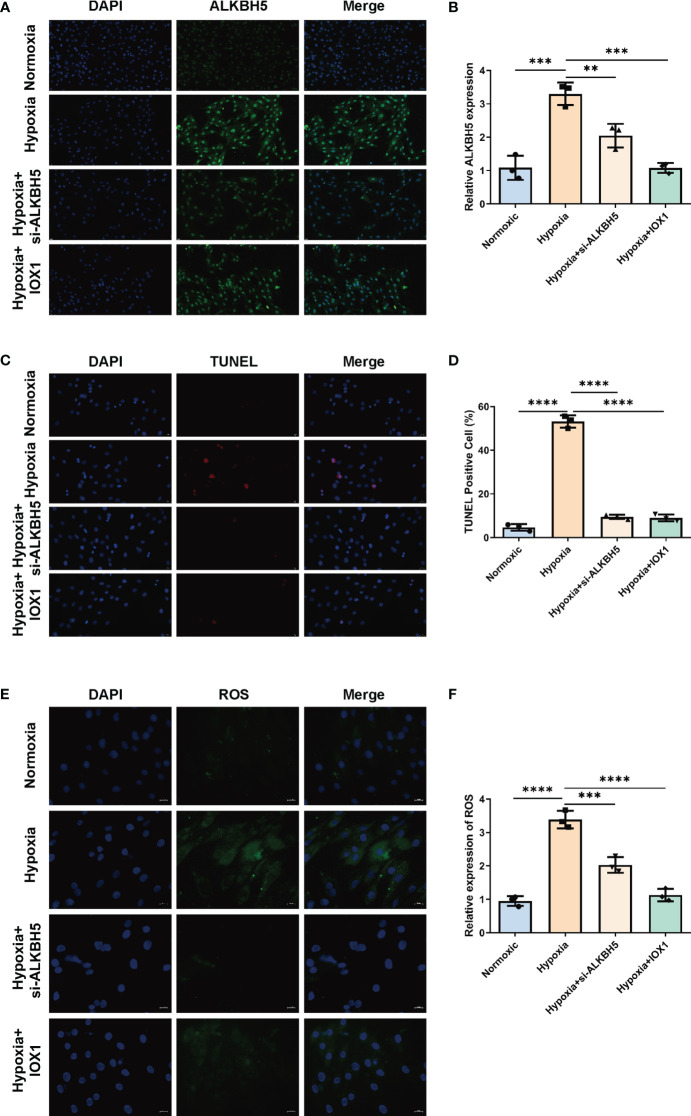
Therapeutic targeting of ALKBH5 attenuates H/R-induced apoptosis and ROS in H9C2 cells. **(A)** Representative immunofluorescent images of ALKBH5 in H9C2 cells with normoxia, hypoxia, hypoxia + ALKBH5 inhibitor IOX1, or hypoxia + si-ALKBH5. Scale bar, 20 μm. **(B)** Quantification of ALKBH5 expression in the above H9C2 cells. **(C)** Representative images of TUNEL staining in H9C2 cells with normoxia, hypoxia, hypoxia + ALKBH5 inhibitor IOX1, or hypoxia + si-ALKBH5. Scale bar, 20 μm. **(D)** Quantification of TUNEL-positive cell percentage in the above cells. **p < 0.01; ***p < 0.001; ****p < 0.0001. **(E)** Representative photographs of DCFH-DA-stained ROS in H9C2 cells with normoxia, hypoxia, hypoxia + ALKBH5 inhibitor IOX1, or hypoxia + si-ALKBH5. Scale bar, 20 μm. **(F)** Quantification of intracellular ROS levels in the above H9C2 cells. ***p < 0.001; ****p < 0.0001.

**Figure 10:**
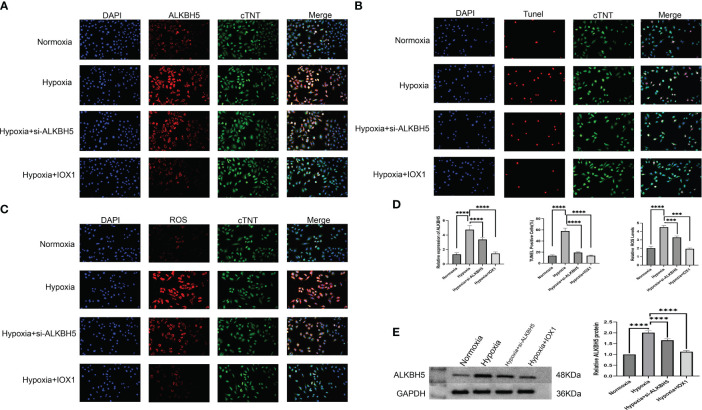
Therapeutic targeting of ALKBH5 alleviates apoptosis and attenuates intracellular ROS of H/R-induced NRCMs. **(A)** Representative immunofluorescent images of ALKBH5 in NRCMs with normoxia, hypoxia, hypoxia + ALKBH5 inhibitor IOX1, or hypoxia +si-ALKBH5. Scale bar, 50 μm. **(B)** Representative images of TUNEL staining in NRCMs with normoxia, hypoxia, hypoxia + ALKBH5 inhibitor IOX1, or hypoxia + si-ALKBH5. Scale bar, 50 μm. **(C)** Representative photographs of DCFH-DA-stained ROS in NRCMs with normoxia, hypoxia, hypoxia + ALKBH5 inhibitor IOX1, or hypoxia + si-ALKBH5. Scale bar, 50 μm. **(D)** Quantification of ALKBH5 expression in NRCMs, quantification of TUNEL-positive cell percentage in NRCMs, and quantification of intracellular ROS levels in NRCMs. ***p < 0.001; ****p < 0.0001. **(E)** Representative Western blot images and quantification of ALKBH5 protein levels in NRCMs. ***p < 0.001; ****p < 0.0001.

We further focused on the role of m^6^A regulator ALKBH5 in AMI. We established an H/R-induced H9C2 cell model and an NRCM model. ALKBH5 expression was greatly increased in hypoxic H9C2 cells and in the NRCM model in comparison to normoxia (**Figures 9A, B**, **10A, D**). To downregulate ALKBH5 expression, H9C2 cells and the NRCM model were pretreated with the ALKBH5 inhibitor IOX1 or transfected with si-ALKBH5. As expected, ALKBH5 expression was notably suppressed in hypoxic H9C2 cells and in the NRCM model. The WB results also show the same trend (**Figure 10E**). TUNEL staining showed that H9C2 cell and NRCM apoptosis was significantly enhanced by hypoxic conditions (**Figures 9C, D**, **10B, D**). Therapeutic targeting of ALKBH5 by its inhibitor IOX1 or si-ALKBH5 greatly ameliorated hypoxia-induced cell apoptosis.

### Therapeutic targeting of ALKBH5 attenuates intracellular ROS in H/R-induced H9C2 cells and NRCMs

Based on the DCFH-DA staining method, we measured the influence of ALKBH5 on intracellular ROS levels. It was found that intracellular ROS levels were prominently enhanced in hypoxic H9C2 cells and NRCMs versus the normoxic condition (**Figures 9E, F**, **10C, D**). The ALKBH5 inhibitor IOX1 and si-ALKBH5 both remarkably alleviated intracellular ROS levels in hypoxic H9C2 cells and NRCMs.

### ALKBH5 is highly expressed in myocardial infarction models and is associated with myocardial fibrosis and cardiomyocyte apoptosis

**Figure 11:**
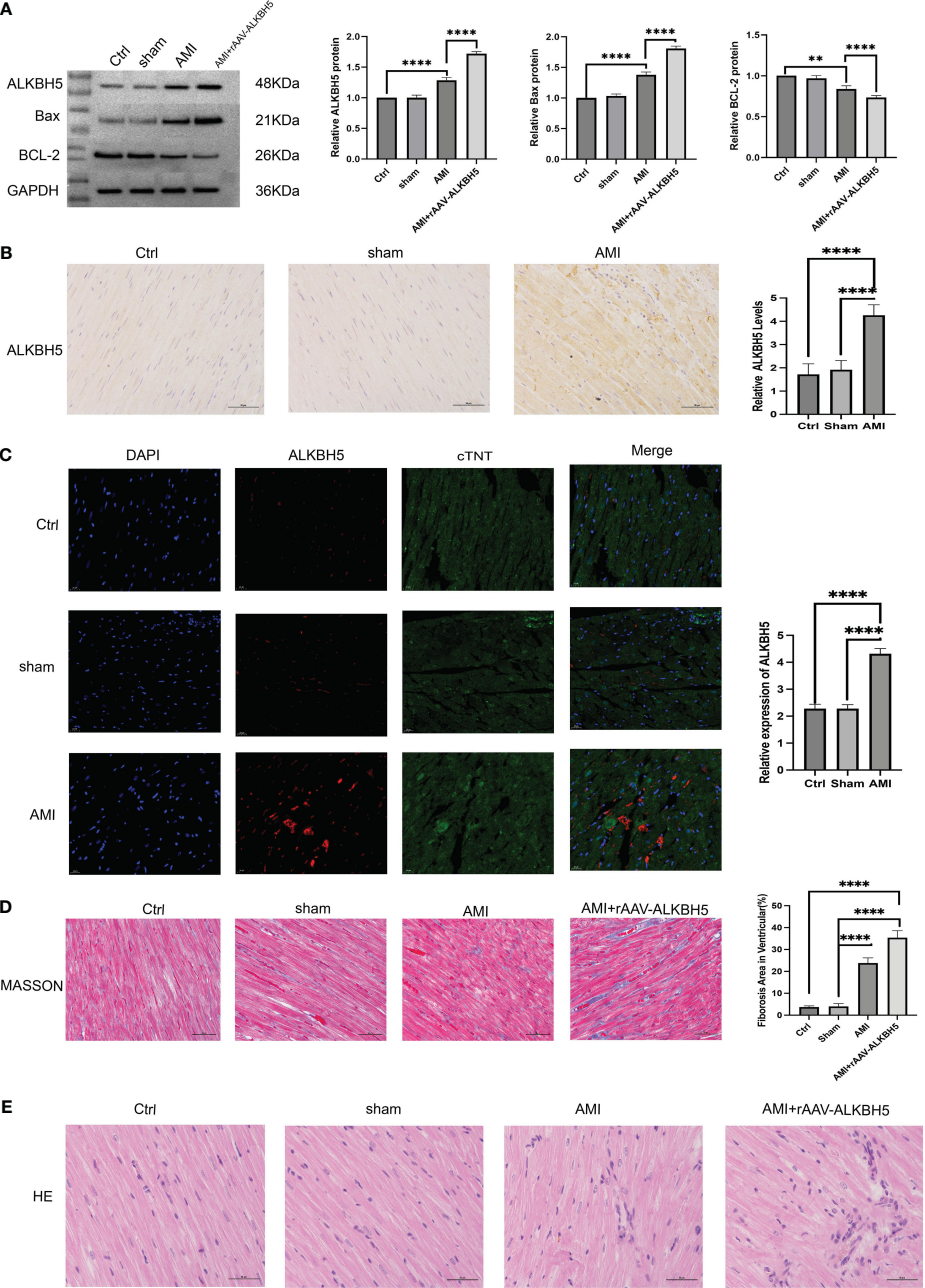
ALKBH5 is highly expressed in myocardial infarction models and is associated with myocardial fibrosis and cardiomyocyte apoptosis. **(A)** Representative Western blot images and quantification of ALKBH5, Bax, and BCL-2 protein levels in the heart tissue of the control, sham operation, AMI, or AMI+rAAV-ALKBH5 groups. ****p < 0.0001. **(B)** Representative images of immunohistochemical ALKBH5 staining in control, sham operation, and AMI hearts, quantified at the right. ****p < 0.0001. Scale bar, 50 μm. **(C)** Representative immunofluorescent images of ALKBH5 in the heart tissue of the control, sham operation, or AMI groups, quantified at the right. Scale bar, 50 μm. ****p < 0.0001. **(D)** Representative micrographs of Masson staining in the heart tissue of the control, sham operation, AMI, or AMI+rAAV-ALKBH5 groups. Scar tissue and viable myocardium are identified in blue and red, respectively. Quantifications of the fibrotic areas are shown on the right. Scale bar, 50 μm. ****p < 0.0001. **(E)** Representative images of H&E staining of the hearts from the four groups of mice. Scale bar, 50 μm.

We then concentrated on examining the function of the m6A modulator ALKBH5 in AMI. We established an AMI animal model. ALKBH5 expression was greatly increased in AMI mice in comparison to the control and sham operation groups. To overexpress the ALKBH5 level, mice were injected with rAAV-ALKBH5. As expected, ALKBH5 expression was notably overexpressed in AMI mice (**Figures 11A-C**). Masson and H&E staining showed that the fibrosis level was higher in the AMI and AMI+rAAV-ALKBH5 groups than in the control and sham operation groups. Especially in the AMI+rAAV-ALKBH5 group, the fibrosis level was the highest (**Figures 11D, E**). As for apoptosis, Bax and BCL-2 were used to evaluate the apoptosis level. The results demonstrated that the expression of Bax was the highest in the AMI+rAAV-ALKBH5 group and significantly elevated in both the AMI+rAAV-ALKBH5 and AMI groups compared with the control and sham surgery groups. Conversely, BCL-2 exhibited a completely opposite trend, with its lowest expression observed in the AMI+rAAV-ALKBH5 group. This indicated that the level of cardiomyocyte apoptosis is the highest in the AMI+rAAV-ALKBH5 group (**Figure 11A**).

## Discussion

Deregulation of epigenetic processes and aberrant gene expression are crucial mechanisms in AMI (33). Among the RNA modifications, m^6^A represents the most studied RNA epi-transcriptomic modification (34). Mounting evidence proves that m^6^A modification affects AMI initiation and progression (35). Consistently, our research uncovered that deregulated m^6^A regulators occurred in circulating cells of AMI, with upregulated ELAVL1, YTHDF1, IGF2BP1, IGFBP2, RBM15B, IGFBP1, and ALKBH5 as well as downregulated FMR1, LRPPRC, YTHDC1, RBMX, HNRNPA2B1, YTHDC2, RBM15, CBLL1, METTL16, ZC3H13, YTHDF3, WTAP, and FTO.

Early diagnosis and targeted management are essential for AMI patients’ prognostic outcome. Predictive models have proven to be valuable in increasing tailored care through heightening informed decision-making (36). By incorporating 10 key m^6^A regulators (METTL16, WTAP, RBM15, CBLL1, YTHDC1, FMR1, IGFBP1, RBMX, ELAVL1, and ALKBH5), we proposed an m^6^A regulator-based gene signature that can diagnose AMI with strong sensitivity and specificity. Additionally, an m^6^A regulator-based nomogram was defined for risk prediction. To facilitate precision medicine, AMI patients were further categorized into three m^6^A clusters. Each cluster presented a unique m^6^A modification pattern, revealing the disease heterogeneity among AMI patients. In addition, we proposed the m^6^A score for individually quantifying m^6^A modification levels in AMI patients. Among the key m^6^A regulators, it was proven that suppressing ALKBH5 could alleviate apoptosis and intracellular ROS levels in H/R H9C2 cells.

Inflammatory response intimately contributes to AMI, which requires the coordinated efforts of diverse immune cells (37). The heterogeneity in immune cells existed across m^6^A clusters. Among m^6^A regulators, FMR1 presented the strongest positive association with activated CD4^+^ T cells, with the strongest negative relationship between LRPPRC and MDSCs. Moreover, the m^6^A score exhibited positive relationships with activated CD4^+^ and CD8- T cells and immature dendritic cells, with negative correlations to MDSCs, macrophages, monocytes, natural killer T cells, and type 1 helper cells. Thus, m^6^A modification might mediate the immune microenvironment of AMI (38).

Oxidative stress is an imbalance state between oxidation and antioxidation (39). NFE2L2 (NRF2), a key mediator of oxidative stress, possessed the highest transcript level in m^6^A cluster C, followed by clusters B and C, while the m^6^A score was positively associated with NFE2L2. Several experimental studies unveiled that m^6^A modification impacts distinct pathophysiological processes through modulating NRF2-mediated oxidative stress (40–42). Ferroptosis influences the activity of glutathione peroxidase by inducting small molecules, resulting in membrane lipid peroxidation because of redox imbalance and excess ROS generation, destroying the integrity of the cell membrane (43). Iron accumulation in the endoplasmic reticulum contributes to ferroptosis in myocardial ischemia–reperfusion damage (44). The three m^6^A clusters presented distinct expression levels of ferroptosis genes, with prominent relationships of m^6^A score with ferroptosis genes, indicating the functional roles of m^6^A in modulating ferroptosis during AMI. Despite this, prospective cohorts are required to prove the diagnostic efficacy of m^6^A regulators. Additionally, the regulatory mechanisms of m^6^A on immune cell infiltration, ferroptosis, and oxidative stress in AMI will be conducted through experimental validation.

## Conclusion

Collectively, our research unveiled the deregulation of m^6^A regulators during AMI. We defined an m^6^A regulator gene signature that accurately diagnosed and predicted the risk of AMI. In addition, AMI patients were classified into three m^6^A clusters with diverse m^6^A modification patterns. The m^6^A score was computed to quantify m^6^A modification levels. The regulatory functions of m^6^A modification in immune cell infiltration, ferroptosis, and oxidative stress were also investigated during AMI. Therapeutic targeting of ALKBH5 notably ameliorated apoptosis and intracellular ROS in H/R-induced H9C2 cells and NRCMs, revealing the potential of ALKBH5 as a treatment target of AMI. Altogether, our findings demonstrated that regulation of m^6^A modification might result in AMI progression and support future studies in the clinically relevant and hopeful field.

## Data availability statement

The datasets presented in this study can be found in online repositories. The names of the repository/repositories and accession number(s) can be found in the article/**Supplementary Material**.

## Ethics statement

The studies involving humans were approved by the Ethics Committee of the People’s Hospital of Xinjiang Uygur Autonomous Region. The studies were conducted in accordance with the local legislation and institutional requirements. The participants provided their written informed consent to participate in this study.

## Author contributions

PC: Data curation, Formal Analysis, Methodology, Software, Writing – original draft. XZ: Data curation, Formal Analysis, Methodology, Software, Writing – original draft, Investigation, Validation, Visualization. LZ: Data curation, Methodology, Software, Writing – original draft. YW: Methodology, Validation, Visualization, Writing – original draft. MW: Software, Visualization, Writing – original draft. GA: Validation, Writing – original draft. XC: Conceptualization, Investigation, Supervision, Writing – review & editing. YY: Conceptualization, Investigation, Supervision, Writing – review & editing.

## Supplementary material

Supplementary Figure 1 Verification of the diagnostic efficacy of key m^6^A regulators in the GSE48060 dataset. (A-J) ROCs of each key m^6^A regulator in diagnosing AMI.

Supplementary Table 1 The genomic location of m6A regulators were visulaized through circos plot.

Supplementary Table 2 The information of m^6^A cluster-relevant genes.
