# Supplementary figures and images for: Characterization of the m6A regulators’ landscape highlights the clinical significance of acute myocardial infarction
Source: Front Immunol. 2024 Mar 20;15:1308978. doi: 10.3389/fimmu.2024.1308978 (PMC10987706; doi:10.3389/fimmu.2024.1308978)

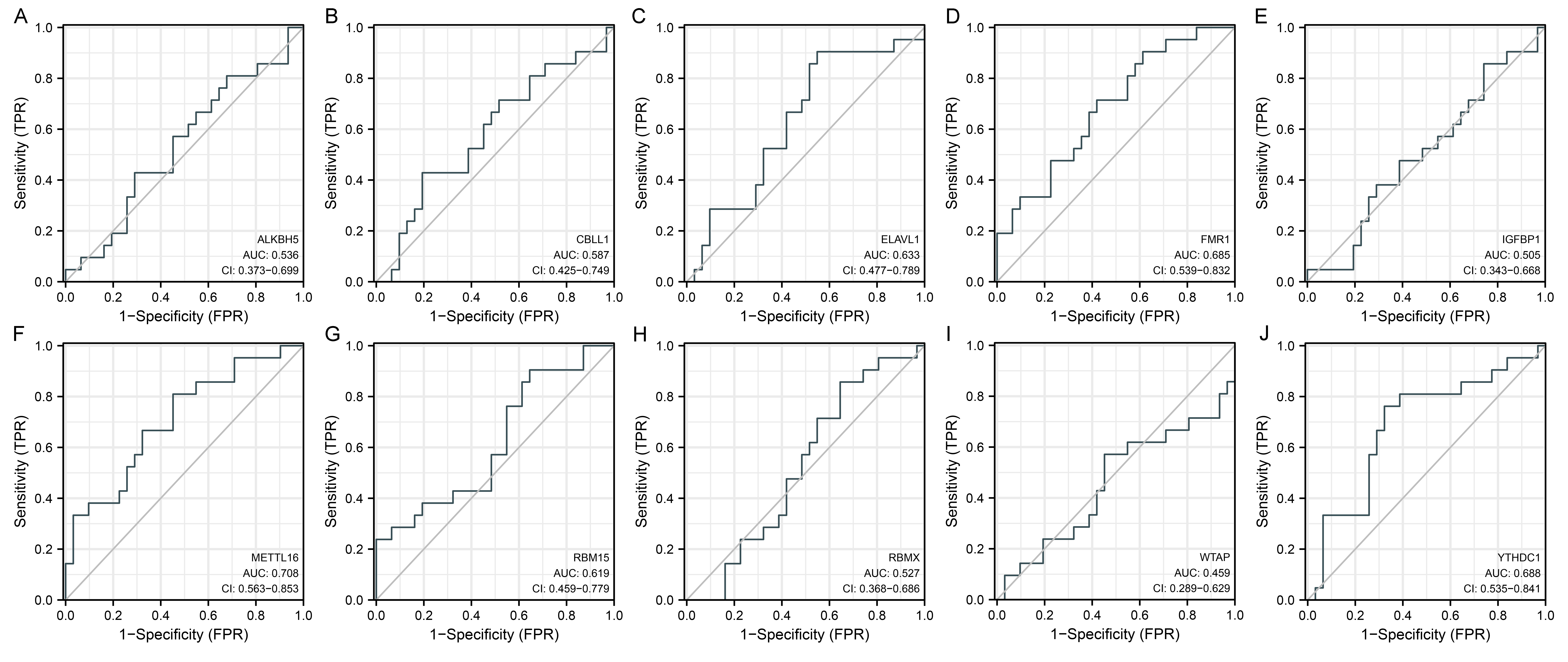

Supplement: Supplementary Figure 1 — Verification of the diagnostic efficacy of key m6A regulators in the GSE48060 dataset. (A-J) ROCs of each key m6A regulator in diagnosing AMI. [file Image_1.tif]
